# SERPINA3: A novel inflammatory biomarker associated with cerebral small vessel disease burden in ischemic stroke

**DOI:** 10.1111/cns.14472

**Published:** 2023-09-18

**Authors:** Xiao Hu, Zhong‐Song Xiao, Yi‐Qing Shen, Wen‐Song Yang, Peng Wang, Pei‐Zheng Li, Zi‐Jie Wang, Ming‐Jun Pu, Li‐Bo Zhao, Peng Xie, Qi Li

**Affiliations:** ^1^ Department of Neurology The First Affiliated Hospital of Chongqing Medical University Chongqing China; ^2^ NHC Key Laboratory of Diagnosis and Treatment on Brain Functional Diseases The First Affiliated Hospital of Chongqing Medical University Chongqing China; ^3^ Department of Neurology The Second Hospital of Anhui Medical University Hefei China; ^4^ Department of Neurology Yongchuan Hospital of Chongqing Medical University Chongqing China; ^5^ Chongqing Key Laboratory of Cerebrovascular Disease Research Chongqing China

**Keywords:** cerebral small vessel disease, inflammation, SERPINA3, white matter hyperintensity

## Abstract

**Background and Objective:**

Inflammation has emerged as a prominent risk factor for cerebral small vessel disease (CSVD). However, the specific association between various inflammatory biomarkers and the development of CSVD remains unclear. Serine proteinase inhibitor A3 (SERPINA3), Matrix metalloproteinase‐9 (MMP‐9), Tissue inhibitor metalloproteinase‐1 (TIMP‐1), Monocyte Chemoattractant Protein‐1 (MCP‐1) are several inflammatory biomarkers that are potentially involved in the development of CSVD. In this present study, we aimed to investigate the relationship between candidate molecules and CSVD features.

**Method:**

The concentration of each biomarker was measured in 79 acute ischemic stroke patients admitted within 72 h after symptom onset. The associations between blood levels of inflammatory markers and CSVD score were investigated, as well as each CSVD feature, including white matter hyperintensities (WMH), lacunes, and enlarged perivascular spaces (EPVS).

**Results:**

The mean age was 69.0 ± 11.8 years, and 65.8% of participants were male. Higher SERPINA3 level (>78.90 ng/mL) was significantly associated with larger WMH volume and higher scores on Fazekas's scale in all three models. Multiple regression analyses revealed the linear association between absolute WMH burden and SERPINA3 level, especially in model 3 (*β* = 0.14; 95% confidence interval [CI], 0.04–0.24; *p* =0.008). Restricted cubic spline regression demonstrated a dose–response relationship between SERPINA3 level and larger WMH volume (*p* nonlineariy = 0.0366 and 0.0378 in model 2 and mode 3, respectively). Using a receiving operating characteristic (ROC) curve, plasma SERPINA3 level of 64.15 ng/mL distinguished WMH >7.8 mL with the highest sensitivity and specificity (75.92% and 60%, respectively, area under curve [AUC] = 0.668, *p* = 0.0102). No statistically significant relationship has been found between other candidate biomarkers and CSVD features.

**Conclusion:**

In summary, among four inflammatory biomarkers that we investigated, SERPINA3 level at baseline was associated with WMH severity, which revealed a novel biomarker for CSVD and validated its relationship with inflammation and endothelial dysfunction.

## Graphical abstract

Inflammation plays an important role in the pathological mechanism of cerebral small vessel disease (CSVD). We reported for the first time that SERPINA3, an inflammatory molecule, was independently correlated with the severity of white matter hyperintensity, a key feature of CSVD (A). This association was absent in the context of acute infarct volume (B).

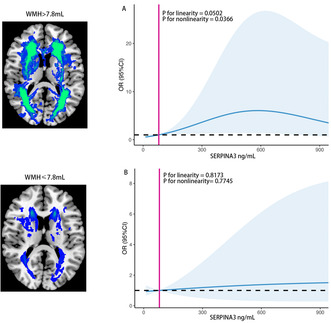

## 1 INTRODUCTION

Cerebral small vessel disease (CSVD) is a group of neurological conditions caused by abnormalities of cerebral arterioles, capillaries, and venules.^1^ It is strongly associated with an elevated susceptibility to stroke, dementia, and depression.^2^ Furthermore, it has been established that CSVD is related to the augmented risk of recurrent vascular events in patients with ischemic stroke (IS).^3^ Additionally, previous reports indicated that CSVD accounts for 25% of strokes.^4^ Despite its considerable influence on public health, preventive treatment of CSVD is still limited due to the unclarity of its mechanisms. CSVD is often characterized by several magnetic resonance imaging (MRI) features including white matter hyperintensity (WMH), lacunes, enlarged perivascular spaces (EPVS), cerebral microbleeds (CMB), and cerebral atrophy.^5^ CSVD‐related MRI markers can be found in approximately 70% of individuals over 65 years and are exacerbated by aging.^6^, ^7^ Previous investigations demonstrated a noteworthy correlation between MRI characteristics of CSVD and inflammatory biomarkers,^8^, ^9^, ^10^ suggesting a fundamental inflammatory process contributing to the pathogenesis of CSVD. However, as there is a wide spectrum of inflammatory markers that could be involved in this pathophysiological process, there is a compelling need for a more in‐depth investigation of the association between diverse inflammatory biomarkers and the characteristic features of CSVD.

In this study, we selected several candidate inflammatory molecules that could potentially be associated with CSVD. Serine proteinase inhibitor A3 (SERPINA3), a member of the serine protease inhibitor superfamily, has been reported to participate in pathological inflammatory processes of numerous neurological diseases such as ischemic stroke,^11^ intracerebral hemorrhage,^12^ and Alzheimer's disease.^13^ However, despite being a potential predisposing factor for ischemic stroke, the relationship between CSVD burden and SERPINA3 has never been investigated in patients with IS. Matrix metalloproteinases (MMPs) are a group of proteolytic enzymes that contribute to endothelial injury of central nervous diseases.^14^ Among them, MMP‐9 is associated with an increased risk of stroke and higher WMH burden.^15^, ^16^ Tissue inhibitor of metalloproteinase‐1 (TIMP‐1), the most potent inhibitor of MMP‐9,^17^ serves as a mediator in various biological functions, including atherosclerosis, apoptosis, angiogenesis, and inflammation.^18^ A small sample‐sized study reported an association between higher TIMP‐1 levels and severe WMH burden, suggesting a possible link with CSVD.^19^ Monocyte chemoattractant protein‐1 (MCP‐1, also known as C‐C motif chemokine ligand 2) is a pro‐inflammatory protein involved in the process of regulating leukocyte recruitment during inflammation.^20^ Elevated MCP‐1 has been correlated with increased long‐term risk of stroke in a population‐based study.^21^ Nevertheless, the evidence of the relationship between these inflammatory biomarkers and CSVD burden in IS patients remains scarce. Here, we performed a cross‐sectional study in a cohort of ischemic stroke patients, the main aim of our study was to investigate whether there's a correlation between those candidate inflammatory biomarkers and specific CSVD features.

## 2 METHODS

### 2.1 Subjects

Acute ischemic stroke patients within 72 h of symptom onset who were admitted to the neurology department of the First Affiliated Hospital of Chongqing Medical University between June 2021 and May 2022 were included in our study. Acute ischemic stroke was defined as foacal or global neurological deficit lasting more than 24 h according to World Health Organization criteria.^22^ Patients were excluded from this study if they met the following criteria: (1) patients who did not undergo MRI examination after admission because of contradictions and other reasons; (2) patients without blood sample collection within 24 h after admission; (3) patients with systemic inflammatory disease, or neoplastic disease. The institutional review board of the First Affiliated Hospital of Chongqing Medical University granted approval for this study. Informed consent was obtained from all participants or their authorized trustees.

Stroke severity was assessed by the National Institute of Health Stroke Score (NIHSS) within 24 h after admission. Demographic data, medical history, vascular risk factors, and laboratory measurements were collected. Stroke etiology was categorized according to Trial of ORG 10172 in Acute Stroke Treatment (TOAST) classification.^23^

### 2.2 Biomarker measurement

Fasting blood sample was drawn on the second morning of admission for biochemical examination. All blood samples were centrifuged within 1 h and divided into aliquots, and then immediately frozen at −80°C. Plasma SERPINA3, MMP‐9, TIMP‐1, and MCP‐1 concentrations were measured using commercially available ELISA kits (FineTest) in duplicate on the same run according to manufacturers' instructions.

### 2.3 Imaging analysis

Brain MRI was performed according to the standardized imaging protocol for acute ischemic stroke in our institution. SVD markers on MRI were ascertained in accordance with Standards for Reporting Vascular Changes on Neuroimaging criteria.^5^ Lacunes were defined as ovoid to round cavities with the same signals as cerebrospinal fluid (CSF) on T2 and FLAIR and ranging in size from 3 mm to 15 mm. White matter hyperintensity (WMH) severity was rated on axial FLAIR images according to the Fazekas scale.^24^ Enlarged periventricular space (EPVS) burden in basal ganglia (BG) and centrum semiovale (CSO) was evaluated by a 4‐level score established by Doubal et al.^25^ Total SVD burden was calculated using a validated score ranging from 0 to 3,^26^, ^27^ which comprises 1 point each for any presence of lacunes, moderate to severe BG‐EPVS (score 2 to 4), and extensive WMH (a Fazekas score of 2–3 in periventricular area or 3 for deep WMH). We dichotomized patients into two groups based on SVD score: none‐to‐mild SVD group (score 0–1) and moderate‐to‐severe group (score 2–3).

CAT12 toolbox (https://neuro‐jena.github.io/cat//index.html) implemented in Statistical Parametric Mapping 12 (SPM12, http://www.fil.ion.ucl.ac.uk/spm/) was utilized to segment gray matter (GM), white matter (WM), and CSF on T1‐weighted images. Total intracrainal volume (TIV) was calculated as the sum of GM, WM, and CSF. MRICron software (https://www.nitrc.org/projects/mricron) was employed to create binary mask of acute ischemic lesion and calculate infarct volume. To measure white matter hyperintensity volume (WMHV), the lesion growth algorithm included in the lesion segmentation tool (LST) toolbox (http://www.statistical‐modelling.de/lst.html) for SPM automatically segmented WMH on FLAIR images and then was manually inspected and corrected by a trained neurologist.

### 2.4 Statistical analysis

Categorical variables are expressed as frequencies and percentages, quantitative variables are presented as mean ± standard deviations (SD) or median and interquartile range (IQR), contingent upon the results of normality assessment using Kolmogorov–Smirnov test. For the comparison between none‐mild SVD group and moderate–severe SVD group, student's *t*‐test (for normally distributed variables) or Mann–Whitney *U* test (for non‐normally distributed variables) were used for continuous variables, and categorical variables were compared with *χ*2 or Fisher's exact tests. Plasma concentration of each biomarker was dichotomized into two levels (≥median and <median), ordinal or binary logistic regression models were applied to examine their association with total CSVD burden and each SVD feature (WMH burden, EPVS burden, presence of lacune). Three models were conducted for each CSVD feature. In model 1, only age and sex were adjusted. In model 2, we adjusted covariates including age, baseline NIHSS, current drinking, current smoking, vascular risk factors including hypertension, diabetes, and toast classification. In model 3, age, baseline NIHSS, hypertension, diabetes, infarct volume, and triglyceride (TG), previous stroke, and toast classification were adjusted. Variables with a VIF >5 were excluded. In addition, multiple linear regression models were built to determine relationship between white matter volume and biomarker concentrations (divided into quartiles), respectively. To measure absolute WMH burden, we defined normalized WMH volume (nWMHV) as WMH volume in native space divided by TIV and expressed in percent, then log‐transformed to reach a normal distribution, *β* coefficient and 95% confidence interval (CI) was calculated. Receiver operating characteristic curve (ROC) analysis was used to calculate the cut‐off point of SERPINA3 to discriminate patients in the larger or smaller WMH volume group (threshold = 7.8 mL), based on the maximum value of Youden index. Moreover, we performed a restricted cubic spline regression analysis to assess the association pattern between plasma SERPINA3 and larger WMH volume (>7.8 mL) using a logistic model adjusted for variables in model 2 and model 3, respectively. Three knots were placed at the 10th, 50th, and 90th percentiles of plasma SERPINA3. A *p* of <0.05 was considered to indicate statistical significance. Statistical analyses were carried out with SPSS (version 26) and GraphPad Prism software (version 9).

## 3 RESULTS

### 3.1 Patient characteristics

**FIGURE 1:**
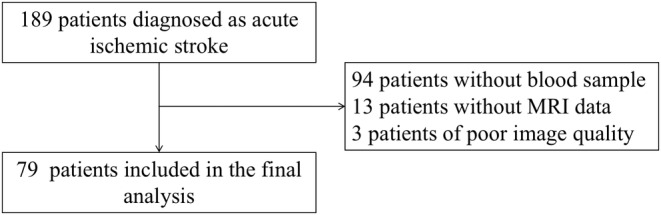
Flowchart of patient selection.

Between December 2020 and April 2022, 189 patients diagnosed with acute ischemic stroke were screened. Of those, 94 patients without available blood sample, 13 patients without MRI data, and 3 patients with poor image quality were exclude. The flowchart of patient selection is shown in Figure 1. A total of 79 ischemic stroke patients with available blood samples were included in our final analysis. Of these patients, 45 had a CSVD score of 0–1, and 34 were classified as moderate‐to‐severe SVD defined as a CSVD score of 2–3. The average age was 69.0 years, and 52 (65.8%) patients were male. The median NIHSS score was 4 (IQR, 2–6) at baseline, and the median infarct volume was 1.9 mL (IQR, 0.5–7.5). Patient characteristics were compared between none‐mild SVD group and moderate–severe SVD group (Table 1). Compared with the none‐mild SVD group, the moderate–severe SVD group had a larger WMH volume (median, 5.2 mL versus 24.4 mL, *p* < 0.001) and was more likely to be large artery artherosclerosis (LAA, *p* = 0.002) according to the TOAST classification. Nevertheless, other baseline variables, including baseline infarct volume (1.7 mL vs. 2.1 mL, *p* = 0.905), as well as vascular risk factors such as hypertension (*p* = 0.485), diabetes (*p* = 0.621), current smoking (*p* = 0.310) and alcohol consumption (*p* = 0.531), were comparable between the two groups.

**TABLE 1:** Baseline characteristics.

	Total (*n* = 79)	None‐Mild SVD group (*n* = 45)	Moderate–Severe SVD group (*n* = 34)	*p* Value
Demographic characteristics				
Age, years, mean (SD)	69.0 (11.8)	66.8 (12.7)	72.0 (9.9)	0.118
Sex, male, *n* (%)	52 (65.8)	31 (68.9)	21 (61.8)	0.509
Admission to blood drawn, h, median (IQR)	20.0 (17.0–23.0)	20.0 (16.5–22.0)	20.0 (17.0–24.0)	0.347
Clinical characteristics				
NIHSS, median (IQR)	4 (2–6)	3 (1–7)	5 (3–6)	0.162
Previous stroke, *n* (%)	4 (5.1)	0 (0.0)	4 (11.8)	0.065
Thrombolysis, *n* (%)	6 (7.6)	2 (4.4)	4 (11.8)	0.431
TOAST classification				
LAA	29 (36.7)	10 (22.2)	19 (55.9)	0.002
SVO	36 (45.6)	25 (55.6)	11 (32.4)	0.040
CE	9 (11.4)	7 (15.6)	2 (5.9)	0.326
SOE	1 (1.3)	0 (0.0)	1 (2.9)	0.430
SUE	4 (5.1)	3 (6.7)	1 (2.9)	0.818
Vascular risk factors				
Hypertension, *n* (%)	50 (63.3)	27 (60.0)	23 (67.6)	0.485
Diabetes mellitus, *n* (%)	21 (26.6)	11 (24.4)	10 (29.4)	0.621
Coronary heart disease, *n* (%)	3 (3.8)	3 (6.7)	0 (0.0)	0.347
Current smoking, *n* (%)	33 (41.8)	21 (46.7)	12 (35.3)	0.310
Current drinking, *n* (%)	19 (24.1)	12 (26.7)	7 (20.6)	0.531
Laboratory findings, median (IQR)				
Glucose, mmol/L	6.1 (5.4–8.3)	6.0 (5.3–8.6)	6.4 (5.6–7.9)	0.458
TG, mmol/L	1.3 (1.0–1.8)	1.4 (1.0–2.4)	1.2 (1.0–1.4)	0.059
TC, mmol/L	4.7 (4.0–5.3)	4.6 (4.0–5.1)	4.8 (4.0–5.5)	0.279
LDL, mmol/L	2.9 (2.2–3.5)	2.9 (1.9–3.4)	2.9 (2.4–3.9)	0.150
HDL, mmol/L	1.1 (1.0–1.4)	1.1 (0.9–1.3)	1.1 (1.0–1.4)	0.367
Hemoglobin, g/L	137 (123–150)	140 (124–153)	134 (123–147)	0.424
WBC count × 10^9^/L	6.7 (5.8–9.2)	6.5 (5.7–8.2)	7.8 (5.8–9.7)	0.398
Neutrophil count × 10^9^/L	5.0 (3.7–7.1)	4.6 (3.7–6.9)	5.0 (3.3–7.3)	0.727
Lymphocyte count × 10^9^/L	1.4 (1.0–1.7)	1.4 (1.0–1.7)	1.4 (1.0–1.8)	0.907
Hs‐CRP, mg/L	2.03 (1.22–6.43)	1.92 (1.28–7.83)	2.85 (1.11–6.43)	0.488
SERPINA3, ng/mL	78.9 (32.3–220.1)	67.4 (23.2–226.2)	108.1 (47.5–217.7)	0.370
MMP‐9, ng/mL	52.6 (28.7–105.4)	57.6 (27.1–103.7)	46.1 (27.7–114.7)	0.628
TIMP‐1, ng/mL	206.5 (103.1–319.6)	207.2 (103.0–352.3)	197.3 (107.5–276.2)	0.464
MCP‐1, pg/mL	58.7 (44.5–86.2)	59.1 (44.6–89.8)	57.2 (43.8–86.7)	0.909
MRI findings				
Infarct volume, mL, median (IQR)	1.9 (0.5–7.5)	1.7 (0.5–7.7)	2.1 (0.5–8.0)	0.905
WMH volume, mL, median (IQR)	7.8 (4.3–22.0)	5.2 (3.0–10.4)	24.4 (6.3–44.0)	<0.001

Abbreviations: CE, cardioembolism; HDL, high density lipoprotein; Hs‐CRP, high‐sensitivity C‐reactive protein; IQR, interquartile range; LAA, large artery atherosclerosis; LDL, low density lipoprotein; MCP‐1, monocyte chemoattractant protein‐1; MMP‐9, matrix metalloproteinase‐9; MRI, magnetic resonance imaging; NIHSS, National Institutes of Health Stroke Scale; SAO, small vessel oclussion; SD, standard derivation; SERPINA3, Serine proteinase inhibitor A3; SOE, stroke of other determined etiology; SUE, stroke of undetermined etiology; SVD, small vessel disease; SVO, small vessel oclussion; TC, total cholesterol; TG, triglyceride; TIMP‐1, tissue inhibitor metalloproteinase‐1; TOAST, Trial of Org 10,172 in Acute Stroke Treatment; WBC, white blood cell; WMH, white matter hyperintensity.

### 3.2 Association between inflammatory biomarkers and CSVD features

The association between each inflammatory biomarker level and CSVD features is illustrated in Table 2. Notably, a higher concentration of SERPINA3 was associated with larger WMH volume after adjusting for age and sex (odds ratio [OR], 2.95; 95% confidence interval [CI], 1.27–6.86; *p* = 0.012). This correlation remained significant in two other statistical models (model 2: OR, 3.37; 95% CI, 1.46–9.24; *p* = 0.006 and model 3: OR, 3.63; 95% CI, 1.41–9.38; *p* = 0.008). The severity of white matter lesions, quantified using the Fazekas Scale, maintained its significance in correlation with SERPINA3 across all three models (model 1: OR, 2.56; 95% CI, 1.13–5.81, *p* = 0.025; model 2: OR, 3.23; 95% CI, 1.33–7.88, *p* = 0.010 and model 3: OR, 3.73; 95% CI, 1.48–9.42, *p* = 0.005). After controlling for age, NIHSS, hypertension, diabetes, infarct volume, triglyceride, previous stroke, and toast classification, an elevated level of SERPINA3 was associated with a higher degree of CSO‐EPVS (OR, 3.06; 95% CI, 1.04–9.01; *p* = 0.042). Nevertheless, an intriguing albeit non‐significant positive trend was unveiled concerning the total CSVD score in model 2 (OR, 2.46; 95% CI, 0.98–6.20; *p* = 0.056). Regarding other inflammatory biomarkers, namely MCP‐1, TIMP‐1, and MMP‐9, our analyses did not reveal any statistically significant correlations between their plasma concentrations and any CSVD feature.

**TABLE 2:** Associations between CSVD features and plasma biomarker concentrations.

	Model 1^a^			Model 2^b^			Model 3^c^		
	OR	95% CI	*p* Value	OR	95% CI	*p* Value	OR	95% CI	*p* Value
CSVD score									
SERPINA3	1.78	0.77–4.10	0.178	2.46	0.98–6.20	0.056	2.33	0.90–5.98	0.080
MCP‐1	0.88	0.38–2.04	0.771	1.34	0.52–3.31	0.537	1.18	0.45–3.09	0.735
TIMP‐1	0.89	0.38–2.09	0.791	1.03	0.42–2.56	0.949	1.24	0.48–3.18	0.655
MMP‐9	0.72	0.32–1.64	0.430	0.87	0.36–2.08	0.751	0.81	0.33–1.99	0.637
Fazekas score									
SERPINA3	2.56	1.13–5.81	0.025	3.23	1.33–7.88	0.010	3.73	1.48–9.42	0.005
MCP‐1	0.73	0.32–1.64	0.445	0.96	0.40–2.27	0.917	1.00	0.40–2.51	0.995
TIMP‐1	0.99	0.44–2.26	0.989	1.22	0.51–2.91	0.655	1.16	0.47–2.87	0.746
MMP‐9	0.90	0.41–2.00	0.796	1.08	0.47–2.50	0.860	1.01	0.42–2.40	0.988
WMH volume (quartile)									
SERPINA3	2.95	1.27–6.86	0.012	3.37	1.46–9.24	0.006	3.63	1.41–9.38	0.008
MCP‐1	0.81	0.35–1.85	0.617	0.97	0.40–2.36	0.951	1.13	0.44–2.92	0.794
TIMP‐1	0.90	0.39–2.07	0.794	0.95	0.39–2.30	0.902	1.12	0.45–2.83	0.806
MMP‐9	0.64	0.29–1.45	0.289	0.70	0.29–1.65	0.410	0.72	0.30–1.76	0.471
CSO‐EPVS degree									
SERPINA3	1.96	0.77–4.97	0.157	2.50	0.88–7.06	0.085	3.06	1.04–9.01	0.042
MCP‐1	0.89	0.35–2.26	0.810	0.95	0.35–2.60	0.916	0.94	0.32–2.80	0.917
TIMP‐1	0.51	0.20–1.33	0.170	0.48	0.17–1.36	0.165	0.54	0.18–1.59	0.262
MMP‐9	1.09	0.44–2.72	0.850	1.44	0.54–3.86	0.470	1.10	0.40–3.04	0.854
BG‐EPVS degree									
SERPINA3	1.57	0.59–4.20	0.370	1.33	0.44–4.02	0.613	2.15	0.67–6.90	0.198
MCP‐1	0.98	0.36–2.62	0.960	1.15	0.37–3.55	0.809	1.61	0.50–5.25	0.426
TIMP‐1	0.82	0.30–2.26	0.705	0.87	0.28–2.74	0.809	0.84	0.26–2.73	0.777
MMP‐9	0.82	0.31–2.17	0.693	0.83	0.27–2.54	0.746	0.92	0.30–2.84	0.882
Presence of lacune									
SERPINA3	1.33	0.53–3.33	0.540	0.85	0.32–2.27	0.746	0.63	0.21–1.93	0.420
MCP‐1	0.64	0.26–1.62	0.346	2.40	0.86–6.68	0.095	1.90	0.57–6.38	0.297
TIMP‐1	0.97	0.38–2.49	0.954	0.93	0.35–2.52	0.892	1.22	0.40–3.73	0.734
MMP‐9	2.42	0.96–6.14	0.062	0.37	0.14–1.02	0.054	0.34	0.11–1.04	0.058

*Note*: plasma concentration of each biomarker was divided into 2 groups (≥median and <median).

Abbreviations: BG, basal ganglia; CI, confidence interval; CSO, centrum semiovale; CSVD, cerebral small vessel disease; EPVS, enlarged perivascular space; MCP‐1, monocyte chemoattractant protein‐1; MMP‐9, matrix metalloproteinase‐9; OR, odds ratio; SERPINA3, Serine proteinase inhibitor A3; TIMP‐1, tissue inhibitor metalloproteinase‐1; WMH, white matter hyperintensity.

a Model 1 adjusted for age and sex.

b Model 2 adjusted for age, baseline NIHSS, current drinking, current smoking, vascular risk factors including hypertension, diabetes, and toast classification.

c Model 3 adjusted for age, baseline NIHSS, vascular risk factors including hypertension, diabetes, infarct volume, and TG, previous stroke and toast classification.

**FIGURE 2:**
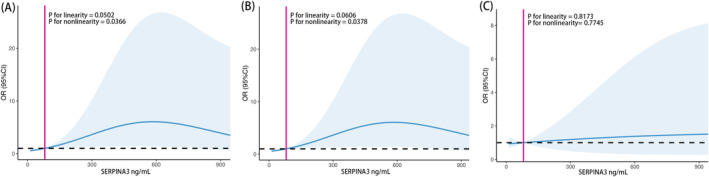
Association between plasma SERPINA3 levels and risk of larger WMH volume (>7.8 mL), and larger infarct volume (>1.9 mL). Adjusted odds ratio (aOR) and 95% confidence interval (CI) derived from restricted cubic regression analysis, three knots were placed at 10th, 50th and 90th percentiles of concentration of SERPINA3. Nonlinear association between larger WMH volume and SERPINA3 concentration adjusted for covariates in model 2 (a) and model 3 (b), respectively. Dose–response relationship between larger acute infarct volume and SERPINA3 concentration (c).

**FIGURE 3:**
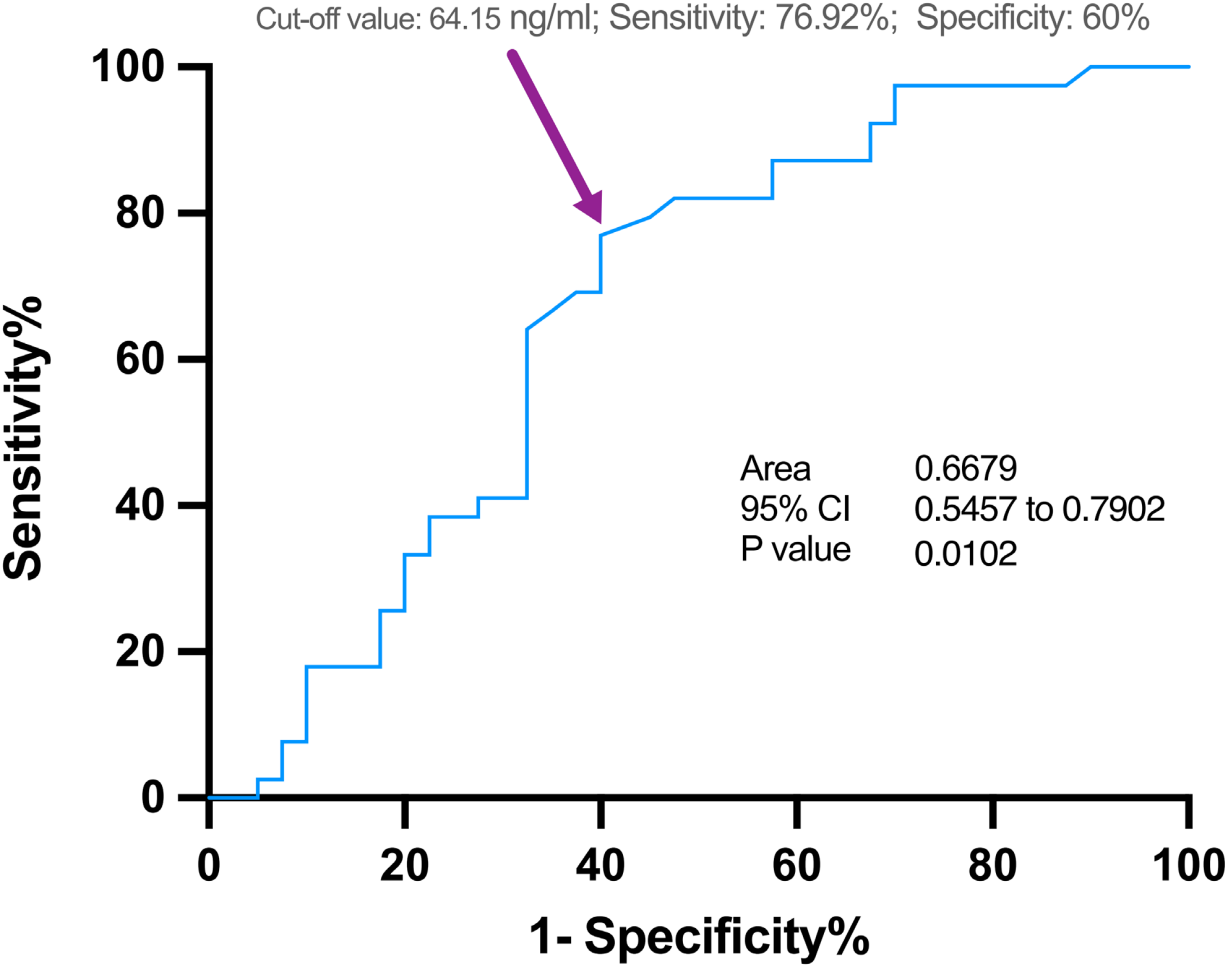
Receiver operator characteristic (ROC) curve demonstrating sensitivity and specificity for differentiating patients in larger WMH volume group based on plasma level of SERPINA3.

In multiple regression analyses, there was a significant positive correlation between SERPINA3 level and normalized WMH volume in model 1 (*β* coefficient = 0.12; 95% CI, 0.02–0.22; *p* = 0.017), model 2 (*β* coefficient = 0.13; 95% CI, 0.04–0.23; *p* = 0.008), and model 3 (*β* coefficient = 0.14; 95% CI, 0.04–0.24; *p* = 0.008), respectively (Table 3). Patients in the third and fourth quartiles of plasma SERPINA3 levels exhibited significantly higher WMH burden in comparison to those in the lowest quartile group. Moreover, results from restricted cubic spline regression analysis were presented in Figure 2, which showed the dose–response relationship between SERPINA3 level and risk of having larger WMH volume (>7.8 mL) in model 2 (*p* for nonlinearity = 0.0366, p for linearity = 0.0502, Figure 2A) and model 3 (*p* for nonlinearity = 0.0378, *p* for linearity = 0.0606, Figure 2B). However, no significant linear or nonlinear association has been found between SERPINA3 level and acute infarct volume (>1.9 mL) after adjusting for age, hypertension, diabetes, smoking, drinking and TOAST classification (*p* for nonlinearity = 0.7745, *p* for linearity = 0.8173, Figure 2C). Furthermore, the sensitivity and specificity of SERPINA3 in the recognition of patients with WMH volume >7.8 mL was plotted in ROC curve with an area under curve (AUC) of 0.668 (*p* = 0.010, Figure 3).

**TABLE 3:** Multiple regression analysis for nWMHV in relation to plasma concentrations of inflammatory biomarkers.

	Beta coefficient and 95% CI					*p* Value
SERPINA3, ng/mL	Q1 (<32.25)	Q2 (32.25–78.90)	Q3 (78.90–220.10)	Q4 (>220.10)		
Model 1^a^	1.0	0.35 (0.04–0.66)	0.49 (0.20–0.79)	0.35 (0.04–0.65)	0.12 (0.02–0.22)	0.017
Model 2^b^	1.0	0.32 (0.05–0.59)	0.52 (0.25–0.79)	0.37 (0.11–0.63)	0.13 (0.04–0.23)	0.008
Model 3^c^	1.0	0.33 (0.06–0.61)	0.51 (0.25–0.77)	0.37 (0.10–0.64)	0.14 (0.04–0.24)	0.008
MCP‐1, pg/mL	Q1 (<44.50)	Q2 (44.50–58.71)	Q3 (58.71–86.16)	Q4 (>86.16)		
Model 1	1.0	−0.08 (−0.41–0.25)	−0.04 (−0.36–0.29)	−0.01 (0.35–0.33)	0.00 (−0.10–0.11)	0.972
Model 2	1.0	−0.09 (−0.38–0.20)	0.02 (−0.28–0.31)	0.05 (−0.27–0.36)	0.05 (−0.84–0.14)	0.640
Model 3	1.0	−0.07 (−0.37–0.23)	0.07 (−0.24–0.37)	0.10 (−0.24–0.43)	0.09 (−0.08–0.16)	0.472
TIMP‐1, ng/mL	Q1 (<103.15)	Q2 (103.15–206.52)	Q3 (206.52–319.64)	Q4 (>319.64)		
Model 1	1.0	−0.14 (−0.46–0.17)	−0.10 (−0.41–0.22)	−0.28 (−0.60–0.04)	−0.16 (−0.18–0.02)	0.123
Model 2	1.0	−0.19 (−0.48–0.11)	−0.13 (−0.42–0.16)	−0.24 (−0.53–0.06)	−0.14 (−0.17–0.04)	0.206
Model 3	1.0	−0.18 (−0.49–0.13)	−0.12 (−0.42–0.19)	−0.24 (−0.54–0.06)	−0.14 (−0.18–0.04)	0.219
MMP‐9, ng/mL	Q1 (<28.65)	Q2 (28.65–52.64)	Q3 (52.64–105.37)	Q4 (>105.37)		
Model 1	1.0	−0.08 (−0.40–0.24)	−0.12 (−0.44–0.21)	0.02 (−0.31–0.34)	0.01 (−0.10–0.11)	0.945
Model 2	1.0	−0.11 (0.42–0.19)	−0.09 (−0.38–0.20)	0.05 (−0.25–0.35)	0.04 (−0.09–0.12)	0.729
Model 3	1.0	−0.10 (−0.41–0.22)	−0.08 (−0.38–0.23)	0.08 (−0.24–0.40)	0.06 (−0.08–0.14)	0.577

Abbreviations: CI, confidence interval; MCP‐1, monocyte chemoattractant protein‐1; MMP‐9, matrix metalloproteinase‐9; nWMHV, normalized white matter hyperintensity; SERPINA3, Serine proteinase inhibitor A3; TIMP‐1, tissue inhibitor metalloproteinase‐1.

^a^ Model 1 adjusted for age and sex.

^b^ Model 2 adjusted for age, baseline NIHSS, current drinking, current smoking, vascular risk factors including hypertension, diabetes, and toast classification.

^c^ Model 3 adjusted for age, baseline NIHSS, vascular risk factors including hypertension, diabetes, infarct volume, and TG, previous stroke and toast classification.

## 4 DISCUSSION

In the present study, we explored the correlations between a range of inflammatory biomarkers and imaging markers of CSVD. Notably, we observed a significant association between elevated SERPINA3 levels and the severity of WMH among patients with acute ischemic stroke. Patients with higher SERPINA3 concentration had larger WMH volume and there was a positive correlation between the plasma level of SERPINA3 and absolute WMH burden. These associations remained significant after adjusting for potential confounding factors. Importantly, our study did not find a linear or nonlinear association between plasma SERPINA3 levels and the volume of acute infarcts.

The role of SERPINA3 in cerebrovascular disease remains controversial. On one hand, SERPINA3 may help reduce the damage caused by ischemic stroke by decreasing apoptosis and neuroinflammation in animal models.^11^ Conversely, recent studies have found that SERPINA3 might be a potential biomarker of poor functional outcome in patients with ICH.^12^ This dual nature of SERPINA3, potentially acting as both a protective agent in ischemic stroke and a risk factor in ICH, underlines the complexity of cerebrovascular system and the diverse roles that a single protein can play within it. In our current investigation, we observed a potential link between SERPINA3 and CSVD, where higher level of SERPINA3 was associated with greater severity of WMH, a prominent imaging hallmark of CSVD. These findings suggest that SERPINA3 may contribute to CSVD burden, a connection that, to our knowledge, has not been previously reported.

CSVD is a significant risk factor for stroke, yet it lacks effective therapeutic strategies due to the complexity of its underlying mechanisms and limited understanding of its etiology.^28^ Cumulative evidence suggested that systemic inflammatory response plays an essential role in cerebral small vessel disease. Among several potential mechanisms, endothelial damage and atherosclerosis are the two most recognized etiologies.^8^, ^10^ However, the broad array of inflammatory biomarkers present challenges for their implementation in the pathophysiological studies of CSVD. These challenges stem from their lack of specificity in chronic arterial degeneration, notably in the case of CRP, IL‐6, TNF‐α,^29^ which are commonly observed molecules in various neurological conditions. In a recent study,^8^ researchers classified 15 serum inflammatory biomarkers into three categories: systemic, endothelial‐related, and media‐related inflammation. The study demonstrated that endothelial‐related inflammatory biomarkers were associated with increased WMH volume and presence of lacunes. These results align with previous findings wherein vascular cell adhesion molecule 1 (VCAM‐1), recognized as a predictor for cerebrovascular disease and a crutial molecule in endothelial damage, was correlated with WMH severity.^30^ As a member of serpin superfamily, SERPINA3 was found to play a critical role in endothelial cell dysfunction in other diseases such as coronary heart disease^31^ and sepsis.^32^ Kim et al reported an elevation in the expression of SERPINA3 as a contributing factor in the blood–brain‐barrier (BBB) dysfunction process, based on their findings in a stem cell model.^33^ Additionally, the expression pattern of SERPINA3 paralleled VCAM‐1, suggesting that SERPINA3 may play a vital role in BBB damage.

In our study, we observed a dose–response relationship between SERPINA3 and WMH severity. Interestingly, this correlation was not present in the context of acute infarct volume, suggesting that SERPINA3 could potentially play a pivotal role in the pathogenesis of WMH and acute ischemic lesion could have little effect on SERPINA3 level. These findings further support a relationship between endothelial dysfunction caused by chronic inflammation and severity of WMH. Consequently, SERPINA3 could potentially serve as a molecular link between inflammation and WMH, offering a new perspective for understanding the pathophysiology of this disease.

Our study exhibits several limitations. Firstly, the small sample size derived from a single center constrains the generalizability of our findings, conclusive investigation of our results demands a large‐scale population‐based study stratified by stroke etiology. Secondly, evaluation of biomarkers in our study was restricted to baseline measurements, precluding investigation into potential temporal variations and their longitudinal prognostic values. Thirdly, the omission of susceptibility weighted imaging (SWI) and gradient recalled echo (GRE) from the routine imaging protocol for ischemic stroke at our hospital precluded the assessment of CMBs. Lastly, the candidate biomarkers included in our analysis are limited. Future studies with large sample size and comprehensive imaging data should explore a broader spectrum of inflammatory biomarkers.

## 5 CONCLUSION

In conclusion, our study unveils a novel correlation between SERPINA3 and WMH severity in a hospital‐based population, which supports the hypothesis that inflammation and endothelial dysfunction contribute to the pathogenesis of CSVD, and SERPINA3 could be an important participant in the development of WMH.

## AUTHOR CONTRIBUTIONS

XH, ZSX, and QL contributed to the conceptualization and design of this study; XH and ZSX contributed to literature review and manuscript drafting; XH and ZJW contributed to statistical analyses; WSY, YQS, PW, PZL, and MJP contributed to data collection; all authors contributed to critical revisions of the manuscript.

## FUNDING INFORMATION

This work was supported by the National Natural Science Foundation of China (No. 82071337).

## CONFLICT OF INTEREST STATEMENT

The authors have no conflicts of interest to disclose.

## Supporting information


Table S1.

## Data Availability

Data are available to researchers upon request for purposes of reproducing the results or replicating the procedure by directly contacting the corresponding author.
