# Supplementary material for: SERPINA3: A novel inflammatory biomarker associated with cerebral small vessel disease burden in ischemic stroke
Source: CNS Neurosci Ther. 2023 Sep 18;30(3):e14472. doi: 10.1111/cns.14472 (PMC10916418; doi:10.1111/cns.14472)
Supplement: Supplementary file 1 — Table S1. [file CNS-30-e14472-s001.docx]

**Supplementary Material**

**Content:** Table S1

**Table S1 Baseline characteristics of original cohort and final cohort**

|  | **Original Cohort**  **(n=189)** | **Final Cohort**  **(n=79)** |
| --- | --- | --- |
| Age, years, mean (SD) | 69.9 (11.5) | 69.0 (11.8) |
| Sex, male, n (%) | 125 (66.1) | 52 (65.8) |
| NIHSS, median (IQR) | 4 (1-11) | 4 (2-6) |
| Previous stroke, n (%) | 38 (20.1) | 4 (5.1) |
| Thrombolysis, n (%) | 23 (12.2) | 6 (7.6) |
| Hypertension, n (%) | 115 (60.8) | 50 (63.3) |
| Diabetes mellitus, n (%) | 64 (33.9) | 21 (26.6) |
| Coronary heart disease, n (%) | 17 (9.0) | 3 (3.8) |
| Current smoking, n (%) | 104 (55.0) | 33 (41.8) |
| Current drinking, n (%) | 42 (22.2) | 19 (24.1) |
| TOAST classification |  |  |
| LAA | 61 (32.3) | 29 (36.7) |
| SVO | 53 (28.0) | 36 (45.6) |
| CE | 37 (19.6) | 9 (11.4) |
| SOE | 2 (1.1) | 1 (1.3) |
| SUE | 12 (6.3) | 4 (5.1) |
| **Laboratory findings, median (IQR)** | | |
| TG, mmol/L | 1.2 (0.9-1.8) | 1.3 (1.0-1.8) |
| TC, mmol/L | 4.5 (3.8-5.2) | 4.7 (4.0-5.3) |
| LDL, mmol/L | 2.8 (2.2-3.4) | 2.9 (2.2-3.5) |
| HDL, mmol/L | 1.1 (1.0-1.4) | 1.1 (1.0-1.4) |
| Hemoglobin, g/L | 135 (123-146) | 137 (123-150) |
| WBC count×10^9^/L | 7.14 (5.85-9.50) | 6.7 (5.8-9.2) |
| Neutrophil count×10^9^/L | 4.8 (3.8-7.3) | 5.0 (3.7-7.1) |
| Lymphocyte count×10^9^/L | 1.4 (1.0-1.8) | 1.4 (1.0-1.7) |
| Hs-CRP, mg/L | 3.1 (1.2-10.1) | 2.03 (1.22-6.43) |

Abbreviations: CE, cardioembolism; HDL, high density lipoprotein; Hs-CRP, high-sensitivity C-reactive protein; IQR, interquartile range; LAA, large artery atherosclerosis; LDL, low density lipoprotein; NIHSS, National Institutes of Health Stroke Scale; SD, standard derivation; SOE, stroke of other determined etiology; SUE, stroke of undetermined etiology; SVO, small vessel oclussion; WBC, white blood cell; TC, total cholesterol; TG, triglyceride; TOAST, Trial of Org 10172 in Acute Stroke Treatment
